# Genomic Tools in Cowpea Breeding Programs: Status and Perspectives

**DOI:** 10.3389/fpls.2016.00757

**Published:** 2016-06-03

**Authors:** Ousmane Boukar, Christian A. Fatokun, Bao-Lam Huynh, Philip A. Roberts, Timothy J. Close

**Affiliations:** ^1^Cowpea Breeding, International Institute of Tropical AgricultureKano, Nigeria; ^2^Cowpea Breeding, International Institute of Tropical AgricultureIbadan, Nigeria; ^3^Department of Nematology, University of California, RiversideRiverside, CA, USA; ^4^Department of Botany and Plant Sciences, University of California, RiversideRiverside, CA, USA

**Keywords:** cowpea, genomics, marker-assisted breeding, *Vigna unguiculata*, blackeye pea

## Abstract

Cowpea is one of the most important grain legumes in sub-Saharan Africa (SSA). It provides strong support to the livelihood of small-scale farmers through its contributions to their nutritional security, income generation and soil fertility enhancement. Worldwide about 6.5 million metric tons of cowpea are produced annually on about 14.5 million hectares. The low productivity of cowpea is attributable to numerous abiotic and biotic constraints. The abiotic stress factors comprise drought, low soil fertility, and heat while biotic constraints include insects, diseases, parasitic weeds, and nematodes. Cowpea farmers also have limited access to quality seeds of improved varieties for planting. Some progress has been made through conventional breeding at international and national research institutions in the last three decades. Cowpea improvement could also benefit from modern breeding methods based on molecular genetic tools. A number of advances in cowpea genetic linkage maps, and quantitative trait loci associated with some desirable traits such as resistance to Striga, Macrophomina, Fusarium wilt, bacterial blight, root-knot nematodes, aphids, and foliar thrips have been reported. An improved consensus genetic linkage map has been developed and used to identify QTLs of additional traits. In order to take advantage of these developments single nucleotide polymorphism (SNP) genotyping is being streamlined to establish an efficient workflow supported by genotyping support service (GSS)-client interactions. About 1100 SNPs mapped on the cowpea genome were converted by LGC Genomics to KASP assays. Several cowpea breeding programs have been exploiting these resources to implement molecular breeding, especially for MARS and MABC, to accelerate cowpea variety improvement. The combination of conventional breeding and molecular breeding strategies, with workflow managed through the CGIAR breeding management system (BMS), promises an increase in the number of improved varieties available to farmers, thereby boosting cowpea production and productivity in SSA.

## Economic importance, growing regions, nutritional value

Globally, cowpea is an important grain legume adapted and grown in dry areas of the tropics and subtropics. In sub-Saharan Africa (SSA), cowpea plays an important role in both human nutritional and food security and income generation for farmers and food vendors. Its grains are rich in protein, carbohydrates and folic acid, and contain respectable amounts of some minerals. Young cowpea leaves are used as spinach in eastern and southern Africa while green immature pods and green mature seeds are also used in Senegal and some other African countries. The most economically important part of the crop remains the dry grain, which is commonly boiled and eaten as beans. The grain can be processed as flour or paste, which is used to make akara (deep-fried) or moin-moin (steamed), eaten as snacks in several western and central African countries. Based on evaluation of 1541 germplasm lines, Boukar et al. (2011) reported that cowpea grains contain on average 25% protein, 53.2 mg/kg iron, 38.1 mg/kg zinc, 826 mg/kg calcium, 1915 mg/kg magnesium, 14,890 mg/kg potassium, and 5055 mg/kg phosphorus. In addition to the grain, the biomass (haulms) from cowpea plants provides important nutritious fodder for ruminants mainly in the Sahel regions of West and Central Africa. Through its ability to fix atmospheric nitrogen, cowpea, like other legumes, contributes to the fertility of soil. Cowpea fixes between 70 and 350 kg nitrogen per hectare and contributes 40–80 kg nitrogen/ha to the soil (Quin, 1995). The estimated potential impact of cowpea research on fixed nitrogen in SSA, for the period 2011–2020 would be about 77,320 tons (CRP GL, 2012).

Although accurate statistics are generally unavailable, cowpea production worldwide is estimated at about 6.5 million metric tons annually on about 14.5 million hectares. About 83% of the global cowpea production is obtained in Africa, with over 80% of African production in West Africa. Nigeria, with an estimated 45% of the world cowpea production and over 55% of the production in Africa, is the world's largest producer and consumer of cowpea, followed by Niger (15%), Brazil (12%), and Burkina Faso (5%). Over the last three decades, global cowpea production grew at an average rate of 5%, with 3.5% annual growth in area and 1.5% growth in yield, and the area expansion accounting for 70% of the total growth during this period (Fatokun et al., 2012b). Globally, the share of cowpea in total area under pulses grew from < 10% in 1990 to nearly 20% in 2007. In West Africa, cowpea occupies over 85% of the area under pulses and 10% of the total cultivated land (Fatokun et al., 2012b). If these past trends in cowpea area expansion and yield continue into the future, the global cowpea supply is projected to reach 9.8 million tons in 2020 and 12.3 million tons in 2030, against the projected global demand of nearly 8.5 million tons in 2020 and 11.2 million tons in 2030. Increased investments in research are needed to generate an increase in yield to meet the projected increasing demand for the crop and to forestall any possible deficit. Through the sales of cowpea products, smallholder farmers in SSA generate some income despite the fact that information about cowpea trade is very scanty. Abate et al. (2012) attributed this lack of information to the limited international trade involving cowpea.

## Production constraints

The production of cowpea is limited by several biotic and abiotic stresses. The biotic stresses include insect pests, diseases, parasitic weeds, and nematodes. At every stage in the life cycle of the crop there is at least one major insect pest that may cause yield losses. Aphid (*Aphis craccivora*) attacks cause the highest amount of damage to the plants mainly at the seedling stage. Flower bud thrips (*Megalurothrips sjostedti*) at flowering stage cause the destruction of flower buds and failure of pod formation. Pods and young shoots are destroyed by pod borers (*Maruca vitrata*) while a complex of pod-sucking bugs (*Clavigralla tomentosicollis, Anoplocnemis curvipes, Nezara viridula*) penetrate and damage seeds in pods. In storage, bruchid weevils (*Callosobruchus maculatus*) cause serious damage to the grains.

In the case of diseases, cowpea is attacked by bacteria, viruses, fungi, and nematodes. Bacterial blight caused by *Xanthomonas vignicola* and bacterial pustule (*Xanthomonas* sp.) are the main bacterial diseases of cowpea. Several viruses infect cowpea including *Cowpea aphid-borne mosaic virus* (CABMV, genus *Potyvirus*, family *Potyviridae*); *Bean common mosaic virus*—blackeye cowpea mosaic strain (BCMV—BlCM, genus *Potyvirus*, family *Potyviridae*); *Cowpea mosaic virus* (CPMV, genus *Comovirus*, family *Secoviridae*); *Southern bean mosaic virus* (SBMV, genus *Sobemovirus*); *Cowpea mottle virus* (CPMoV, genus *Carmovirus*, family *Tombusviridae*); *Cucumber mosaic virus* (CMV, genus *Cucumovirus*, family *Bromoviridae*); *Cowpea mild mottle virus* (CPMMV, genus *Carlavirus*, family *Betaflexiviridae*); and *Cowpea golden mosaic virus* (CGMV, genus *Begomovirus*, family *Geminiviridae*). The main fungi known to cause diseases in cowpea plants include *Colletotrichum* sp. causing anthracnose and brown blotch, *Macrophomina phaseolina* causing charcoal rot, ashy stem blight and stem canker, *Cercospora canescens* causing cercospora leaf spot, *Elsinoe phaseoli* causing scab, and *Rhizoctonia solani* causing web blight. Plant-parasitic nematodes, especially root-knot nematodes (*Meloidogyne* spp.), damage cowpea root systems and cause yield suppression in many cowpea production areas. These diseases may occur singly or in combinations of two or more pathogens, and in some cases are strongly influenced by the growing environment, for example, ashy stem blight caused by Macrophomina is much more prevalent and severe in drought-stressed cowpea plantings.

Parasitic weeds, *Striga gesnerioides* and *Alectra vogelii*, can cause significant damage to cowpea production. Striga is mainly present in the dry savannah areas of West and Central Africa while Alectra is found predominantly in eastern and southern Africa.

Abiotic stresses affecting cowpea production include drought, heat, and low soil fertility. Although the crop is known to be drought tolerant, its yield can be reduced significantly when exposed to seedling, mid-season or terminal drought. Heat may cause serious damage during the off-season cropping. High night temperatures lead to flower abortion thereby preventing pod formation and a consequent reduction in grain yield. Soils that are deficient in phosphorous, an element required for nitrogen-fixation in legume root nodules, may lower the productivity of cowpea.

## Genetic linkage groups, breeding behavior, wild species

Cowpea is a diploid with 2*n* = 22. Thus, there are 11 linkage groups as revealed by several reports on genetic linkage mapping based on molecular markers. More details about the development and current status of cowpea linkage groups are provided in Section Available Genomic Resources of this review.

Cowpea is a highly self-pollinating crop. The extent of outcrossing is therefore low and varies with environment. The development of improved varieties has been mainly through pure line selection, mass selection, pedigree breeding, single-seed descent, and backcross methods. The genetic base of most of the improved varieties that have been released to farmers for planting is narrow. This is no surprise because improved lines are mostly used as parents in developing populations from which new varieties are derived. In the study reported by Li et al. (2001), the level of SSR polymorphism among improved breeding lines was found to be low when compared with those lines generated using newly acquired germplasm lines as parents. The need for varieties with broad genetic base in farmers' fields should be given priority attention in SSA cowpea breeding programs.

Cowpea belongs to the genus *Vigna*, comprised of several sections, species, sub-species, and varieties. Cowpea belongs to section *Catiang*, species *unguiculata*, sub-species *unguiculata*. All cultivated cowpea and its close cross-compatible relatives belong to *Vigna unguiculata* (Marechal et al., 1978). Three wild subspecies of *V. unguiculata*, namely subspecies *dekindtiana* or *spontanea*, subspecies *stenophylla* and subspecies *tenuis* are recognized. Several taxonomists have proposed different subspecies and names for some of the cowpea wild relatives. There appears to be no consensus yet on the proper classification of the cowpea wild relatives and this has complicated efforts to define the primary and secondary gene pools for cowpea. It should be noted that varying levels of success have been achieved in efforts to make crosses between members of sub-species belonging to *V. unguiculata*. Fatokun and Singh (1987) had to apply embryo rescue in order to successfully cross a cultivated cowpea line with the wild relative *V. unguiculata* subspecies *pubescens*. The hybrid that was rescued through *in vitro* culture was partially fertile. In the several crosses that have been made between cowpea and its wild relatives, the hybrids always showed varying levels of partial fertility. Backcrossing of the hybrids to cultivated cowpea should however improve the level of fertility in subsequent generations. A major drawback to the use of wild cowpea relatives in cowpea breeding is the small seed-size associated with the wild forms. Since small seed-size is dominant to large seed several backcrosses are required in order to recover the desired seed-size of the cultivated type. This is necessary because consumers prefer large seed-size. The application of now available molecular marker tools (Table 1) should facilitate progress in rapid recovery of the genome of the cultivated parental lines.

**Table 1:** **Some cowpea genomics resources**.

**Resources**	**Short description**	**Use**	**References**
Physical Map of cowpea	60,000 BACs from IT97K-499-35 were fingerprinted. The final physical map is an assembly of 43,717 BACs with a depth of 11 × genome coverage.	Tool for gene discovery	Close et al., 2011; http://phymap.ucdavis.edu:8080/cowpea/
HarvEST:Cowpea	EST database with gene function analysis and primer design.	Online cowpea genomics browser	Muchero et al., 2009a,b; http://harvest.ucr.edu/
Cowpea Genespace/Genomics Knowledge Base (CGKB)	Genetic markers, gene-space, metabolic pathways, mitochondrial, and chloroplast sequences.	Tool for gene discovery; enzyme and metabolic pathway	Chen et al., 2007; http://cowpeagenomics.med.virginia.edu/CGKB/
The Cowpea Genomics Initiative (CGI)	Some advances in cowpea genomics.	Tools for gene discovery and cowpea improvement	Chen et al., 2007; http://cowpeagenomics.med.virginia.edu/
Microarray chip	41,949 EST sequences from drought stressed and non-stressed drought susceptible and tolerant cowpea materials generated, representing 16,954 unigenes.	For expression analysis in cowpea	Contact S. Hearne, CIMMYT, Mexico, s.hearne@cgiar.org
			The ESTs are all available in Harvest database of cowpea (UCR and GENBANK on NCBI)
Validated SSR marker kit	Reference kit of 20 SSRs used to define the Cowpea Germplasm Reference Set representing the genetic diversity of the entirety of the IITA cowpea germplasm bank collection.	For diversity analysis and gene discovery	Available from Generation Challenge Program, CIMMYT, Texcoco, Mexico http://info@generationcp.org
Cowpea consensus genetic linkage map	A consensus map containing 1107 EST- derived SNP markers (856 bins) on 11 linkage groups (680 cM) was constructed from 13 population-specific maps.	For QTL identification, map-based cloning, diversity, association mapping	Lucas et al., 2011
Software	‘SNP Selector’, ‘KBioConverter’, and ‘Backcross Selector’ used for the management of genotyping data.	For molecular breeding	(http://breedit.org/) and https://www.integratedbreeding.net/

## Target traits

To alleviate the devastations caused by numerous cowpea production constraints, breeding programs in SSA and USA are implementing both molecular and conventional breeding to develop improved lines with high grain yield potential, resistance to biotic stresses, tolerance to abiotic factors, adaptation to major production agro-ecologies, and traits preferred by consumers and producers.

Sources of genes for several of these traits have been identified through screening of the germplasm available in different countries. The International Institute of Tropical Agriculture (IITA) is maintaining in its genetic resources center about 15,000 accessions of cultivated cowpea and more than 2000 wild relatives. Mining these resources has resulted in the identification of several sources of resistance to biotic and abiotic stresses. Several authors have reported on those germplasm lines that are important sources of resistance for use in breeding programs (Ferry and Singh, 1997; Singh, 2002; Boukar et al., 2015).

Sources of new traits continue to be identified in cowpea germplasm, and the traits defined at high genetic resolution with the new genotyping resources available for identifying marker-trait associations as described in more detail in Section Marker-Trait Associations. As recent examples, Souleymane et al. (2013) confirmed the tolerance to aphids of the improved line IT97K-556-6, in which two resistance loci were mapped (Huynh et al., 2015) and also identified a new source of aphid resistance from a cowpea wild relative, TVNu 1158. The cowpea accession “Sanzi” was identified as a source of genes for resistance to flower bud thrips in Nigeria, Mali and Cameroon. A recent screening of about 200 accessions identified TVu 1272 from Uganda and TVu 16514 from Nigeria as resistant to *S. gesnerioides*. Out of 1300 accessions screened for drought tolerance, 20 were identified with higher levels of tolerance than others (Fatokun et al., 2012a). Six of these accessions have been used in multiple crosses including Danila, TVu 557, TVu 1438, TVu 4574, TVu 6443, and TVu 11982. A set of 1541 cowpea germplasm lines were evaluated for the content of protein and minerals (Fe, Zn, Mg, Ca, and K) in grains (Boukar et al., 2011). Lines rich in grain protein included TVu 10425 (32.2%), TVu 2822 (31.8%), TVu 16531 (31.3%), TVu 450 (31.1%), and TVu 16616 (31.0%). Lines exhibiting high levels of Fe included TVu 2723 (79.5 mg/kg), TVu 14878 (79.5 mg/kg), TVu 2852 (78.7 mg/kg), TVu 526 (78.1 mg/kg), and TVu 10342 (77.0 mg/kg). Lines with high zinc content were TVu 10342 (58.0 mg/kg), TVu 1732 (56.1 mg/kg), TVu 9576 (55.3 mg/kg), TVu 2651 (54.5 mg/kg), and TVu 1877 (54.0 mg/kg). Interestingly, lines with high iron content were also rich in zinc and protein content, implying that these desirable minerals could be selected for concurrently in breeding programs to develop nutrient dense improved varieties.

These genetic sources of desirable traits have been used in hybridization programmes to generate several segregating populations, which were used to select plants with good combinations of target traits (high yield potential, resistance to biotic and abiotic stresses, and consumer preferences). Different breeding methods applicable to self-pollinated crops are employed in cowpea genetic improvement including mass selection and pure line breeding, pedigree selection, single seed descent, bulk selection, backcrossing, mutation breeding, and farmer-participatory varietal selection. Generally, combinations or modifications of these breeding methods are also adopted as necessary. More than 20 IITA breeding lines were released in about 10 countries from 2005 to 2015 (Table 2). Many of the varieties combine high grain yield with resistance to Striga and Alectra. An example is the breeding line IT97K-499-35, which has been released in Niger, Nigeria, Ghana, and Mali; many farmers have adopted this line because of its superior performance.

**Table 2:** **List of IITA varieties released from 2005 to 2015 in sub-Saharan Africa**.

**Year of release**	**Variety**	**Country**
2005	IT93K-452-1, IT90K-277-2	Nigeria
2008	IT97K-499-35	Nigeria
2009	IT89KD-288, IT89KD-391	Nigeria
	IT97K-499-35, IT97K-499-38, IT98K-205-8	Niger
2010	IT97K-499-35, IT93K-876-30	Mali
	IT99K-573-1-1	Niger
2011	IT82E-16, IT00K-1263, IT97K-1069-6	Mozambique
	IT99K-494-6	Malawi
	IT99K-573-1-1, IT99K-573-2-1	Nigeria
2012	IT99K-7-21-2-2-1, IT99K-573-1-1	Tanzania
2013	IT99K-573-2-1, IT98K-205-8	Burkina Faso
	IT95K-193-12	Benin
2015	IT00K-1263, IT99K-1122	Tanzania
	IT07K-292-10, IT07K-318-33	Nigeria
	TVx 194801 F, IT05K-321-2, IT97K-390-2, IT82E-16, IT82E-18, IT99K-494-4	Swaziland
	IT99K-573-1-1, IT99K-573-2-1	Sierra Leone

Cowpea is grown mainly for the protein-rich grains for human consumption. There are now cowpea varieties classified as dual purpose because they produce high grain yield as well as high biomass. The biomass of the haulm, which remains after harvest, is a source of quality fodder for ruminant livestock especially in the Sahelian region of SSA. Cowpea is prone to attack by a myriad of insect pests. These insects cause appreciable grain yield reductions if not controlled using insecticides. It is not uncommon for some farmers in the Sahel to make some income selling fodder from their cowpea fields, which may have suffered serious insect damage to grain yield.

*M. vitrata* is a Lepidopteran insect pest of cowpea. It is the most cosmopolitan of cowpea insect pests and farmers need to apply insecticides to their fields to protect their cowpea crop. Efforts had been made to develop cowpea varieties with resistance to this insect but without success, as there are no resistant lines among accessions of cultivated cowpea. Genetic transformation of cowpea was embarked upon in order to obtain Maruca resistant lines. Popelka et al. (2006) reported successful transformation of cowpea with the *Bt* gene that is efficacious against Maruca. Currently efforts are ongoing to transfer the *Bt* gene to cowpea varieties with high grain yield and farmers' and consumers' preferred attributes.

## Available genomic resources

The development of genomic resources for cowpea has been more recent than those developed for many other crops. Most early efforts in cowpea were focused on molecular diversity and genetic linkage mapping. Genetic diversity studies have used different marker systems as technologies have advanced, including allozymes (Panella and Gepts, 1992; Pasquet, 1999, 2000), seed storage proteins (Fotso et al., 1994), chloroplast DNA polymorphism (Vaillancourt and Weeden, 1992), restriction fragment length polymorphism (RFLP) (Fatokun et al., 1993), amplified fragment length polymorphisms (AFLP) (Fatokun et al., 1997; Tosti and Negri, 2002; Fang et al., 2007), DNA amplification fingerprinting (DAF) (Spencer et al., 2000; Simon et al., 2007), random amplified polymorphic DNA (RAPD) (Mignouna et al., 1998; Fall et al., 2003; Nkongolo, 2003; Ba et al., 2004; Diouf and Hilu, 2005; Xavier et al., 2005; Zannou et al., 2008), simple sequence repeats (SSRs) (Ogunkanmi et al., 2008; Uma et al., 2009; Xu et al., 2010), cross species SSRs from Medicago (Sawadogo et al., 2010), inter-simple sequence repeats (Ghalmi et al., 2010), sequence tagged microsatellite sites (STMS) (Choumane et al., 2000; Li et al., 2001; Abe et al., 2003; He et al., 2003), and single nucleotide polymorphism (SNP) markers (Huynh et al., 2013). Reports from these studies provide information about origins, taxonomy, domestication, and patterns of genetic variation of cowpea. The development of genome resources in cowpea is now progressing with marker technology advancement.

Linkage mapping provides a framework for downstream analyses including quantitative trait loci (QTL) identification, map-based cloning, diversity analysis, association mapping, and molecular breeding (Lucas et al., 2011). The first linkage map for cowpea was developed using a mapping population of 58 F_2_ plants derived from a cross between IT84S-2246-4 and TVNu 1963 (Fatokun et al., 1993). The map had 89 loci including 79 RFLP, five RAPD and four cDNA markers as well as one simply inherited morphological trait. These markers were distributed on 10 linkage groups that spanned 680 cM of the cowpea genome. Menendez et al. (1997) developed the second cowpea genetic linkage map using 94 F_8_ recombinant inbred lines (RILs) derived from a cross between two cultivated genotypes IT84S-2049 and 524B. A total of 181 loci, comprising 133 RAPDs, 19 RFLPs, 25 AFLPs and three each of morphological and biochemical markers were assigned to 12 LGs spanning 972 cM with an average distance of 6.4 cM between markers. This second map was improved with the addition of 242 new AFLP markers, which generated 11 LGs spanning a total of 2670 cM, with an average distance of 6.43 cM between markers (Ouédraogo et al., 2002a). A third genetic map was developed using 94 F_8_ RILs derived from the cross between an improved cultivated cowpea line, IT84S-2246-4, and a wild relative (*V. unguiculata* spp. *dekindtiana* var. *pubescens)* TVNu 110-3A (Ubi et al., 2000). This map consisted of 80 mapped loci (77 RAPD and 3 morphological loci) spanning 669.8 cM of the genome making 12 LGs with an average distance of 9.9 cM between marker loci. With the development of an Illumina GoldenGate Assay, a SNP consensus map with 928 SNP markers on 619 unique map positions distributed over 11 LGs, covering a total genetic distance of 680 cM was established based on the genotyping of 741 members of six bi-parental RIL populations derived from the following crosses: 524B × IT84S-2049, CB27 × 24-125B-1, CB46 × IT93K-503-1, Dan Ila × TVu-7778, TVu-14676 × IT84S-2246-4, and Yacine × 58-77 (Muchero et al., 2009a). This first consensus map resulted in a resolution of 0.73 cM average distance between two adjacent markers or 1 SNP per 668 kbp considering the cowpea genome to be 620 Mbp. The resolution of this consensus genetic map was improved by genotyping 579 individuals from additional populations consisting of five RILs (from UCR–US, IITA–Nigeria, ISRA–Senegal, ZAAS–China) and two F4 populations (Lucas et al., 2011). This new map contained 33% more bins (856), 19% more markers and had an improved order compared to the first consensus genetic map. Updated versions of cowpea consensus maps are accessible via HarvEST:Cowpea (http://harvest.ucr.edu/). Now that linkage maps for cowpea with this marker density are available, there are increased opportunities for QTL resolution, map-based cloning, assessment of genetic diversity, association mapping, and marker-assisted breeding.

The genetic linkage maps that have been published for cowpea are based mainly on molecular markers which are not yet aligned with physical cowpea chromosomes. However, synteny has been reported between cowpea and mung bean (*Vigna radiata*; Menancio-Hautea et al., 1993) based on RFLP derived separate maps of both crops. These authors also reported that 90% of the RFLP probes they tested hybridized with both cowpea and mung bean. Some RFLP markers that mapped in both crops were found to be co-linear on linkage groups of the two crops. Lucas et al. (2011) also reported that 941 of 1107 total SNP markers i.e., 85% that mapped in cowpea show homologs with soybean (*Glycine max*). The markers also showed synteny and co-linearity in the soybean genome.

### Marker-trait associations

Several linkage maps have been used to identify QTLs for desirable traits in cowpea (Table 3). Omo-Ikerodah et al. (2008) used a cowpea linkage map of AFLP and SSR markers to identify QTLs for resistance to flower bud thrips. Gioi et al. (2012) identified and validated a QTL for cowpea yellow mosaic virus (CYMV) resistance using SSR markers. Molecular markers linked to *S. gesnerioides* race-specific resistance genes in cowpea were reported in different studies. Ouédraogo et al. (2001, 2002b) identified three AFLP markers that are tightly linked to the gene designated *Rsg2–1* which confers resistance to Race 1 of *S. gesnerioides* in Burkina Faso. The AFLP markers were: E-AAC/M-CAA_300_ (2.6 cM), E-ACT/M-CAA_524_ (0.9 cM), and E-ACA/M-CAT 140/150 (0.9 cM) and they mapped to the lower portion of LG1 published by Menendez et al. (1997). These scientists also reported the identification of six AFLP markers [E-ACA/M-CAG_120_ (10.1 cM), E-AGC/M-CAT_80_ (4.1 cM), E-ACA/M-CAT_150_ (2.7 cM), E-AGC/M- CAT_150_ (3.6 cM), E-AAC/M-CAA_300_ (3.6 cM), and E- AGC/M-CAT_70_ (5.1 cM)] mapped to LG6 and associated with resistance to *Striga* Race 3 (SG3) from Nigeria. Two of the AFLP markers were associated with resistance to both *Striga* Races 1 and 3. To facilitate the use of these AFLPs, Ouédraogo et al. (2002a) converted one of these markers to a SCAR (sequence-characterized amplified region) that has been used as an effective and reliable marker in selection for resistance to *Striga* Races 1 and 3. Boukar et al. (2004) identified two AFLP markers closely linked to resistance to *Striga* Race 3 from Nigeria, and converted one (E- ACT/M-CAC_115_ located 4.8 cM from the resistance locus) to a SCAR marker to facilitate its use in breeding programs.

**Table 3:** **Mapping of some cowpea traits**.

**Trait**	**Population**	**Type**	**Marker type**	**No. markers/QTLs**	**Locations**	**PV %**	**References**
Cowpea golden mosaic virus	IT97 K-499-35 × Canapu T16	F_2_	AFLP	3	Same linkage group		Rodrigues et al., 2012
*Striga* resistance	TVx 3236 × IT82D-849	F_2_	AFLP	3	LG1		Ouédraogo et al., 2001
	Tvu 14676 × IT84S-2246–4	F_2_	AFLP	6	LG 1		Ouédraogo et al., 2001
	IT84S-2246 × Tvu14676; TVx 3236 × IT82D-849	F_2_	SCAR (61R and 61R-M2)	2	LG 1		Ouédraogo et al., 2012
	IT93 K-693-2 × IAR1696	F_2_	AFLP/SCAR	4/1	Same linkage map		Boukar et al., 2004
Cowpea bacterial blight resistance	DanIla × TVu7778	RIL	SNP	3	LG3, LG5, LG9	10–22	Agbicodo et al., 2010
Drought-induced senescence	IT93K503–1 × CB46	RIL	AFLP	10	LG1, LG2, LG3, LG5, LG6, LG7, LG9, LG10	5–24	Muchero et al., 2010
Maturity	IT93K503–1 × CB46	RIL	AFLP	2	LG7, LG8	25–29	Muchero et al., 2010
Foliar thrips	CB46 × IT93 K-503-1 and CB27 × IT82E-18	RILs	SNP	3	LG2, LG4 and LG10	9–32	Lucas et al., 2012
Foliar thrips	CB46 × IT93 K-503-1 and CB27 × IT82E-18	RILs	AFLP	3	LG 5 and 7	9–32	Muchero et al., 2010
Hastate leaf shape	Sanzi × Vita 7	RIL	SNP	1	LG 15	74.7	Pottorff et al., 2012a
Seed size	524B × 219-01	RIL	SSR	6	LG1, LG10	9–19	Andargie et al., 2011
Pod fiber layer thickness	524B × 219-01	RIL	SSR	4	LG1, LG10	6–17	Andargie et al., 2011
Pod length	(JP81610 × TVnu457) × JP81610	BC_1_F_1_	SSR	9	LG1, LG2, LG3, LG4, LG5, LG7, LG8, LG9, LG11	31	Kongjaimun et al., 2012a
Domestication-related traits	(JP81610 × JP89083) × JP81610	BC_1_F_1_	SSR	1–11 for most traits	LG3, LG7, LG8, LG11	3–57	Kongjaimun et al., 2012b
Seed weight	IT2246-4 × TVNuI963	F_2_	RFLP	2	LG 2 LG6	37–53	Fatokun et al., 1992
Seed weight	524B × 219-01	RIL	SSR	6	LG1, LG2, LG3, LG10	8–19	Andargie et al., 2011
Charcoal rot resistance	IT93 K-503-1 × CB46	RIL	SNP/AFLP	9	LG2, LG3, LG5, LG6, LG11	8–40	Muchero et al., 2011
Flower and seed coat color	ZN016 × Zhijiang 28-2	RIL	SNP and SSR	1 each	LG8	–	Xu et al., 2011
Time of flower opening	524 B × 219-01	RIL	SSR	5	LG1	9–30	Andargie et al., 2013
Days to flower	524 B × 219-01	RIL	SSR	3	LG1	6–19	Andargie et al., 2013
Days to first flowering	ZN016 × ZJ282	RIL	SNP	3	LG11, LG10, LG3	10–32	Xu et al., 2013
Nodes to first flower	ZN016 × ZJ282	RIL	SNP	4	LG11, LG4, LG2, LG6	11–22	Xu et al., 2013
Pod number per plant	ZN016 × ZJ282	RIL	SSR	3	LG3, LG2, LG4	11–20	Xu et al., 2013
Leaf senescence	ZN016 × ZJ282	RIL	SNP	2	LG11, LG3, LG7	11–29	Xu et al., 2013
Floral scent compounds	524 B × 219-01	RIL	SSR	63	LG1, LG2, LG4	60	Andargie et al., 2014
Heat tolerance	CB27 × IT82E-18	RIL	SNP	5	LG2, LG7, LG6, LG10, LG3	12–18	Lucas et al., 2013a
Seed size	Eight different populations	RILs	SNP	10	LG5, LG7, LG2, LG6, LG8, LG10	47	Lucas et al., 2013b
*Fusarium* wilt resistance (Fot race 3)	CB27 × 24-125B-1	RIL	SNP	1	LG6	28	Pottorff et al., 2012b
*Fusarium* wilt resistance (Fot race 4)	IT93K-503-1 × CB46,	RIL	SNP	1	LG8	19–47	Pottorff et al., 2014
	CB27 × 24-125B-1	RIL	SNP	1	LG9	32–40	Pottorff et al., 2014
	CB27 × IT82E-18	RIL	SNP	1	LG3	18–27	Pottorff et al., 2014
Pod tenderness	(JP81610 × JP89083) × JP81610	BC_1_F_1_	SSR	3	LG 7, LG8, LG11	6–50	Kongjaimun et al., 2013
Pod tenderness	JP81610 × JP89083	F_2_	SSR	2	LG 7, LG8	6–45	Kongjaimun et al., 2013

*PV % represents ranges of phenotypic variation of the given QTLs. Adapted and updated from (Abhishek et al., 2014)*.

In the genetic map published by Ouédraogo et al. (2002a), some resistance genes and biochemical characteristics were mapped. Blackeye cowpea mosaic potyvirus (B1CMV) and southern bean mosaic virus (SBMV) resistance were mapped to LG8 and LG6, respectively, and resistance to cowpea mosaic virus (CPMV) and cowpea severe mosaic virus (CPSMV) were mapped to opposite ends of LG3. The CPSMV resistance mapped near a locus conferring resistance to Fusarium wilt. Ouédraogo et al. (2002a) also mapped resistance to root-knot nematode to one end of LG1 on their genetic map. The biochemical trait dehydrin protein, found in cowpea seed and associated with chilling tolerance at emergence, was mapped to LG2. Agbicodo et al. (2010) identified three QTLs for bacterial blight resistance: *CoBB-1, CoBB-2*, and *CoBB-3* on linkage groups LG3, LG5, and LG9, respectively. The genetic map developed by Ubi et al. (2000) also positioned QTLs for several agronomic and morphological traits, including days to flower, days to maturity, pod length, seeds/pod, leaf length, leaf width, primary leaf length, primary leaf width, and derived traits such as leaf area and primary leaf area.

Two unlinked regions of the cowpea genome carry QTLs explaining 52.7% of variation in seed weight while four unlinked regions of mung bean carry QTLs accounting for 49.7% of variation in the same trait (Fatokun et al., 1992). These authors further reported that in both cowpea and mung bean the QTL regions with strong effects on seed weight were spanned by same RFLP markers in the same linkage order. Their study thus suggests that this genomic region of cowpea and mung bean has remained conserved in both crops through evolution. The earlier-developed genetic maps described here require additional reconciliation with the new SNP-based linkage maps for positioning key trait determinants.

Recently, SNP-based linkage maps have been used to map several additional traits. From a RIL population developed from a cross between IT93K-503-1 (tolerant) and CB46 (susceptible) differing in their tolerance to seedling-stage drought, 10 QTLs were identified (Muchero et al., 2009b). Some of these QTLs coincided with QTLs for stem greenness (*stg*) and recovery dry weight (*rdw*) after drought stress under greenhouse and field conditions. The 10 QTLs were located on LG1, 2, 3, 5, 6, 7, 9, and 10 and accounted for 4.7–24.2% of the phenotypic variance (R^2^). Using the same RIL population, Muchero et al. (2010) identified nine QTLs, accounting for 6.1–40.0% of the phenotypic variance (R^2^) for resistance to Macrophomina using plant mortality data from 3 years of field experiments and disease severity scores from two greenhouse experiments. QTL *Mac-1* was located on LG2, *Mac-2, Mac-3*, and *Mac-4* on LG3, *Mac-5* on LG11, *Mac-6* and *Mac-7* on LG5, and *Mac-8* and *Mac-9* on LG6. This number of QTLs and the relatively low contribution of individual loci suggest a quantitative nature of Macrophomina resistance. Pottorff et al. (2012a) identified a major QTL affecting cowpea leaf shape, which may also influence tolerance to drought. More recently, using phenotypic data from 13 experiments conducted across four countries, Muchero et al. (2013) identified SNP-trait associations based on linkage disequilibrium association mapping, with bi-parental QTL mapping as a complementary strategy. Seven QTLs were associated with stay-green and five of these loci exhibited evidence suggesting pleiotropic effects between delayed senescence, biomass, and grain yield. Among the five putative stay-green QTLs, *Dro-1, Dro*-3, and *Dro*-7 were identified in both RILs and diverse germplasm, each spanning 3.2 cM or less, suggesting that they may be valuable targets for marker-assisted breeding. Targeting subsets of loci with higher additive effects in marker-assisted breeding would enhance drought tolerance and Macrophomina resistance in economically important cultivars.

For heat stress, Pottorff et al. (2014) identified three QTLs, *Hbs-1, Hbs-2*, and *Hbs-3*, associated with heat-induced browning of seed coats using the cowpea RIL populations derived from IT93K-503-1 × CB46 and IT84S-2246 × TVu 14676. The identification of SNP markers co-segregating with the heat-induced browning of seed coats phenotype in the *Hbs-1* and *Hbs-3* loci will help indirect selection in breeding cowpea with better quality grain. In addition, the study revealed ethylene forming enzyme as a cowpea candidate gene for the *Hbs-1* locus and an ACC synthase 1 gene as a cowpea candidate gene for the *Hbs-3* locus.

For cowpea insects, Muchero et al. (2009b) identified three QTLs for resistance to foliar thrips. *Thr-1, Thr-2*, and *Thr-3*, were identified on linkage groups 5 and 7 accounting for 9.1–32.1% of the phenotypic variance. In addition, these authors reported that the peaks of these QTLs are respectively co-located with AFLP markers ACC-CAT7, ACG-CTC5, and AGG-CAT1 that could be used in marker-assisted selection for resistance against foliar thrips. These QTLs were subsequently positioned on a SNP consensus map by Lucas et al. (2012). Huynh et al. (2015) identified one major and one minor QTL conferring aphid resistance on LG 7 and LG 1, respectively, with both of the favorable alleles contributed by IT97K-556-6. The major QTL appeared dominant in a related F2 population. SNP markers flanking each QTL are being used to introgress resistance alleles from IT97K-556-6 into susceptible varieties using marker-assisted backcrossing.

A major QTL conferring resistance to root-knot nematodes has been mapped on linkage group 11 of different mapping populations (Huynh et al., 2016). Root-knot nematodes can be a component of disease complexes with other root pathogens such as Fusarium wilt and root rots. Through their efforts to develop *Fusarium oxysporum* f. sp. *tracheiphilum* resistant cowpea lines, Pottorff et al. (2012b) mapped a *Fot* race 3 resistance locus (*Fot3-1*) to a 1.2 cM region and identified SNP marker 1_1107 as co-segregating with *Fot3-1*. Pyramiding QTLs for resistance to root-knot nematodes and fusarium wilt in breeding programs could enable development of cowpea varieties with healthy root systems.

### Genome sequence efforts in cowpea

The genetic map is being used to anchor an initial whole-genome shotgun (WGS) assembly of cowpea accession IT97K-499-35, which includes sequences for about 97% of all known cowpea genes. Genomic DNA from the reference genotype was shotgun sequenced to ~65 × coverage (one 5-kb library included) using Illumina paired-end technology on GAII, and then assembled together with Sanger BAC-end sequences and “gene-space” sequences (Timko et al., 2008) using SOAPdenovo (Luo et al., 2012). The assembly contains 323,048,341 bp of non-N sequences, with an N50 of 6322. These sequences may be searched by BLAST via harvest-blast.org and downloaded via harvest-web.org. A physical map was developed from BAC libraries of IT97K-499-35 by high information content fingerprinting and computational assembly. This physical map is accessible through http://phymap.ucdavis.edu/cowpea/ and is in the process of being linked to the genetic map and genome sequence. Ongoing work to improve the genome assembly using sequenced BACs, long-read shotgun sequencing and optical mapping is in progress, with a goal of 11 pseudomolecules and an average scaffold length of 56 Mb.

## The use of genomic tools in breeding programs

**Figure 1:**
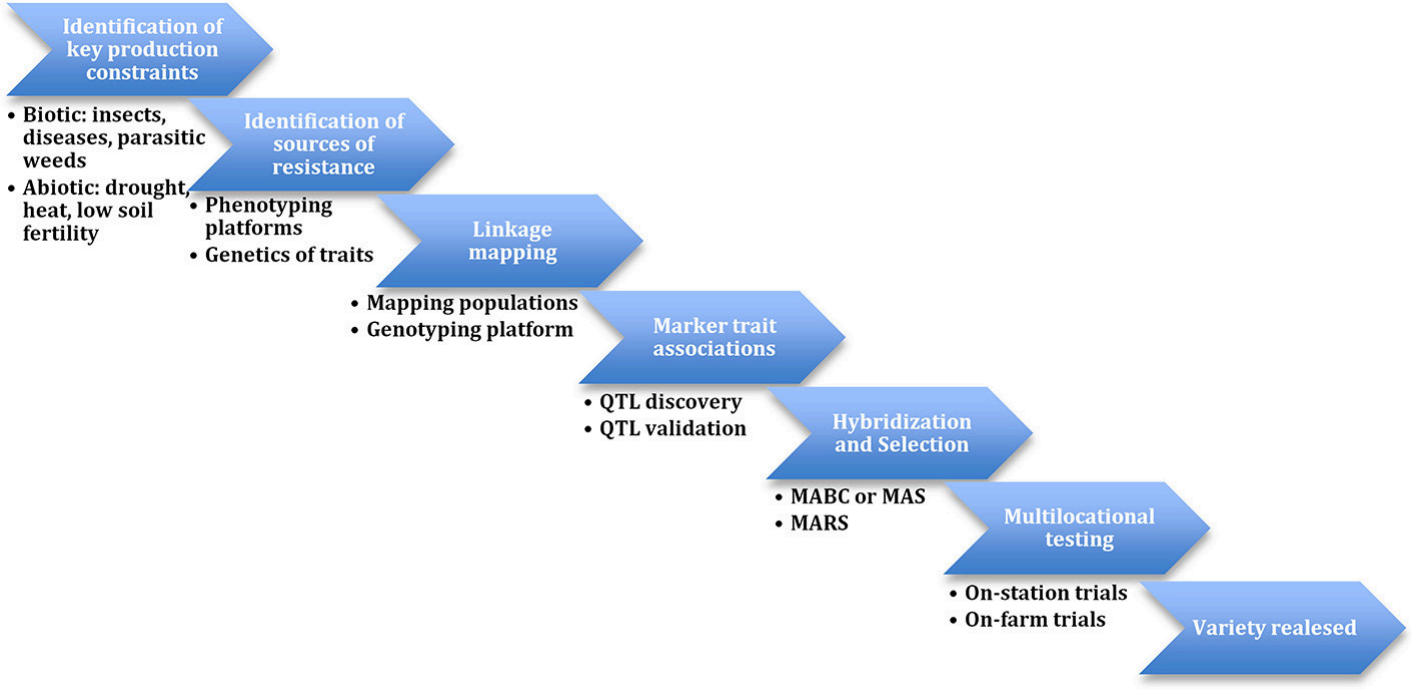
**Scheme of molecular breeding in cowpea**.

During the implementation of the Tropical Legumes I (TLI) project, resources for SNP genotyping and QTL-based selection were developed and applied to marker-assisted recurrent selection (MARS) and marker-assisted backcrossing (MABC) populations (Figure 1). Collaboration between the co-authors, others in the Generation Challenge Program team and LGC Genomics led to conversion of SNP assays to the KASP system. This included 1022 mapped SNPs. Other support tools include the improved cowpea consensus genetic maps (version 4 in Lucas et al., 2011) and version 6 in HarvEST:Cowpea at http://harvest.ucr.edu/). Development of software including “SNP Selector,” “KBioConverter,” and “Backcross Selector” (http://breedit.org/) supported the management of genotyping data. The Breeding Management System (BMS) of the Integrated Breeding Platform (IBP) (https://www.integratedbreeding.net/) is currently facilitating the efficient implementation of cowpea molecular breeding.

MARS lines under development were selected from the populations derived from crosses between elite parents in 2010: (1) Suvita 2 × IT97K-499-35 (Burkina Faso), (2) IT84S-2246 × IT98K-1111-1 (Nigeria), (3) CB27 × IT97K-499-35 (Mozambique), and (4) IT93K-503-1 × Mouride (Senegal). These MARS projects aim to accelerate development of lines carrying all identified QTLs in the homozygous favorable allele state. However, different logistical issues occurred from one location to another. MARS lines with the highest molecular scores for target QTLs were tested in the 2014 main season at INERA, Burkina Faso where two cycles of intercross were performed using 164 SNPs polymorphic between the two parents. The traits of interest include grain yield, drought tolerance, Striga resistance and Macrophomina resistance. At IITA, Nigeria, 102 SNPs were used in the development of MARS populations. With the first cycle of intercrosses completed, about 177 plants were fixed for favorable alleles at seven QTLs affecting yield, drought, and staygreen. Advanced breeding lines are being generated and will be tested across several locations in the 2016 main cropping season. At Eduardo Mondlane University (EMU), Mozambique, large seed, grain quality, and tolerance to heat have been the target traits. MARS lines with favorable seed types and fixed for favorable alleles at QTLs are being screened under drought and irrigated conditions. At ISRA, Senegal, about 136 SNPs well distributed across the cowpea genome and polymorphic between the two parents were used. The target traits have been drought tolerance and resistance to Striga, nematodes and Macrophomina. Adjustments that were necessitated by logistical matters resulted in F2:F6 lines being developed and evaluated across locations in Senegal.

Marker-based backcrossing incorporating foreground and background selection has been used to develop lines that are improved versions of local cultivars carrying target traits or QTLs from donors. At INERA, lines Moussa and KVx745-11P are being improved for Striga resistance and seed size, respectively. In the case of Moussa, the donor of Striga resistance was IT93K-693-2, while for KVx745-11P the donor of seed size was KVx414-22. At IITA, IT93K-452-1, and IT89KD-288 are released varieties that are being improved for Striga resistance, and the donor used is IT97K-499-35. At EMU, IT85F-3139 is being improved using CB27 as donor for grain quality and INIA-41 as donor for nematode resistance and drought tolerance. At ISRA, Melakh is being improved using IT97K-499-39 as the donor of Striga resistance. Most MABC populations were developed up to BC3F4. MABC lines carrying donor alleles and the highest recurrent parent background were evaluated in INERA during the 2014 main season. Other programs are at different levels of seed increase for field evaluations in 2016.

There have also been efforts within the West African Cowpea Consortium (WACC), funded by Kirkhouse Trust, targeting the development by MABC of new cowpea varieties with resistance to the parasitic weed *S. gesnerioides* in six countries.

In Senegal, ISRA at Bambey is applying MAS in breeding new resistant varieties of cowpea to *Striga*, aphid, and *Macrophomina* root rot. In Mali, the Institut d'Economie Rurale (IER) at Bamako is using molecular breeding to develop cowpea lines resistant to the two prevalent strains of Striga present in Mali. In Burkina Faso, INERA at Ouagadougou is using MAS to develop cowpea varieties with resistance to Striga and possessing farmer's preferred traits in different ecological zones. The project has reported the release of four varieties with Striga resistance to farmers in Burkina Faso. Efforts are continuing to apply MAS to develop Striga-, aphid-, and *Colletotrichum capsicii*-resistant cowpea lines. In Ghana, the Savanna Agricultural Research Institute (SARI) at Tamale is using MAS to introgress aphid resistance into known Striga resistant varieties. In Nigeria, the University of Agriculture Makurdi (UAM) is using MAS to develop varieties resistant to Striga, Alectra, aphid, and Fusarium wilt. In Cameroon, the Institut de la Recherche Agronomique pour le Developpement (IRAD) at Maroua is also targeting the development of Striga, aphid, and thrips resistant varieties using MAS.

## Perspectives

Progress in the development of genomic resources for cowpea was achieved recently through the CGIAR Generation Challenge Programme's (GCP) “Tropical Legumes I” project. A high-throughput SNP genotyping platform was established through this project (Muchero et al., 2009a). This platform genotypes simultaneously 96 DNA samples at 1536 SNP loci. Using this, a consensus genetic map was established, which provides the opportunity of determining marker positions with some precision across the cowpea genome. This will facilitate marker-trait association analyses needed for marker-assisted breeding. Currently, efforts are underway to improve both the robustness of the genotyping and the utility of the consensus genetic map through a Feed the Future project entitled, “Innovation Lab for Climate Resilient Cowpea.” Fingerprinting of cowpea breeding programs' preferred accessions are ongoing using nearly 50,000 SNPs. As described above in the genomic resources section, SNP markers and QTLs have been identified for some key biotic and abiotic stresses. Molecular breeding approaches have been initiated in some cowpea breeding programs using LGC Genomics, which converted about 1100 mapped SNPs for use with the KASP platform. Efforts will continue to generate more trait-linked markers, which are intended for breeding applications. The availability of facile genotyping platforms, which may proceed by outsourcing, will accelerate QTL discovery for important traits of cowpea. We anticipate that cowpea breeders will use molecular breeding routinely for the foreseeable future to harness important traits from wild and non-adapted cowpea accessions available in genetic resources centers. Through the implementation of modern breeding, improved lines with higher yield potential may be developed more efficiently.

To increase success, cowpea breeding programs need to address additional challenges, the most significant of which is phenotyping. Phenotyping approaches need to be high-throughput, cost-effective, and precise. Data handling and analysis, and decision support tools such as the BMS of the IBP need to be available to and utilized by cowpea breeders. Also, as discussed by others (Varshney et al., 2014), we must integrate training across scientific fields, including genetics, plant breeding, computer science, mathematics, engineering, biometrics and bioinformatics, and evolve new forms of communication and professional organizations so that genomics-assisted breeding can achieve its potential.

## Summary

Cowpea is one of the most important grain legumes in SSA. It provides strong support to the livelihood of small-scale farmers through its contributions to their nutritional security, income generation and soil fertility enhancement. Worldwide about 6.5 million metric tons of cowpea are produced annually on about 14.5 million hectares. The low productivity of cowpea is attributable to numerous abiotic and biotic constraints. The abiotic stress factors comprise drought, low soil fertility, and heat while biotic constraints include insects, diseases, parasitic weeds, and nematodes. Cowpea farmers also have limited access to quality seeds of improved varieties for planting. Some progress has been made through conventional breeding at international and national research institutions in the last three decades. Cowpea improvement could also benefit from modern breeding methods based on molecular genetic tools. A number of advances in cowpea genetic linkage maps, and QTL associated with some desirable traits such as resistance to Striga, Macrophomina, Fusarium wilt, bacterial blight, root-knot nematodes, aphids, and foliar thrips have been reported. An improved consensus genetic linkage map has been developed and used to identify QTLs of additional traits. In order to take advantage of these developments SNP genotyping is being streamlined to establish an efficient workflow supported by genotyping support service (GSS)-client interactions. About 1100 SNPs mapped on the cowpea genome were converted by LGC Genomics to KASP assays. Several cowpea breeding programs have been exploiting these resources to implement molecular breeding, especially for MARS and MABC, to accelerate cowpea variety improvement. The combination of conventional breeding and molecular breeding strategies, with workflow managed through the CGIAR BMS, promises an increase in the number of improved varieties available to farmers, thereby boosting cowpea production and productivity in SSA.

## Author contributions

OB and CF have contributed in the design, write up, review, and approval of the final version of the manuscript. BH has worked on sections Marker-Trait Associations and The Use of Genomic Tools in Breeding Programs, reviewed and approved the final version of the manuscript. TC has provided information in Section Genome Sequence Efforts in Cowpea. In addition, PR and TC have edited, reviewed, and approved the final version of the manuscript.

### Conflict of interest statement

The authors declare that the research was conducted in the absence of any commercial or financial relationships that could be construed as a potential conflict of interest. The reviewer MKP and handling Editor declared their shared affiliation, and the handling Editor states that the process nevertheless met the standards of a fair and objective review.
